# Lifor Solution: An Alternative Preservation Solution in Small Bowel Transplantation

**DOI:** 10.1155/2016/3925751

**Published:** 2016-01-12

**Authors:** Mingxiao Guo, Chunlei Lu, Ying Gao, Haifeng Zhang, Dongfeng Chen, Yousheng Li

**Affiliations:** ^1^Department of Laparoscopic Surgery Center, Linyi People's Hospital, Shandong University, Linyi 276000, China; ^2^Department of Surgery, Jinling Hospital, Nanjing University School of Medicine, Nanjing 210002, China

## Abstract

*Background and Objectives*. The intestinal mucosa is extremely sensitive to ischemia. Better intestinal preservation is the first step to improve the results of intestinal transplantation. The aim of the study is to investigate the effect of cold Lifor solution on preservation of swine small bowel. *Methods*. Swine ileum segments (200 cm) were allotransplanted heterotopically after 9-hour cold storage with UW solution (group 1, *n* = 6), with Lifor solution (group 2, *n* = 6), or without storage (group 3, *n* = 6), respectively. After cold storage, mucosal adenosine triphosphate (ATP) concentrations and histopathologic analysis after preservation were performed. At day 7 after the transplantation, intestinal absorptive function was also observed. *Results*. After 9 h cold preservation, pathological changes, the content of ATP in the intestinal mucosa, and the intestinal absorptive function after transplantation in group 2 were similar to those of group 1. *Conclusion*. The effect of cold storage of swine small bowel with Lifor solution is similar to that of UW solution. It may provide additional rationale for further exploration of Lifor as an alternative preservation solution in small bowel transplantation.

## 1. Introduction

Intestinal transplantation (ITx) has become an established therapeutic option to patients with irreversible intestinal failure (IF), when complications or failure of parenteral nutrition appears [1]. Recently, advancement in surgical technique, the monitoring and diagnosis of rejection, and the development of new immunosuppressive schedule have significantly increased patient and graft survival rates [2, 3]. However, early postoperative complications, such as endotoxemia, bacterial translocation, and graft dysfunction, which are observed in some cases, may be attributed to hypothermic preservation and ischemia-reperfusion (IR) injury.

Currently, vascular washout and cold storage (CS) with University of Wisconsin solution (UWS) are considered the gold standard for preservation of abdominal organs [4]. This regime effectively protects kidney, pancreas, and liver but less sufficiently protects intestinal integrity and function [5–7]. The lack of an adequate strategy to preserve the intestinal graft allows only a short (6–9 h) preservation span and results in variable degrees of tissue injury limiting the clinical success of ITx [8]. Therefore, better intestinal preservation is a first step to improve the results of ITx.

Lifor is a new type of organ preservation solution comprised of different nutrients, growth factors, and nutrient carriers [9, 10]. Recently, Lifor has been shown to well preserve pig hearts in a low-flow perfused system for up to 20 h and to mitigate both warm and cold renal IR in the rat model, thereby improving renal perfusion [9–12]. However, the effect of Lifor in protecting swine small bowel from IR injury under cold conditions is still unknown.

Therefore, the aim of the study is to examine whether Lifor solution is still much more effective than UW in cold preserving small bowel by a model of swine segmental small bowel allotransplantation.

## 2. Materials and Methods

### 2.1. Animals

Domestic crossbred pigs of either sex, weighing 20 kg to 25 kg, were used in the study after a 5- to 7-day acclimatization. Food and water were provided ad libitum. Animals were treated humanely by use of protocols that were approved by the Institutional Animal Use and Care Committee of Nanjing University.

All animals were randomly divided into three groups. The grafts were preserved for 9 hours at 4°C using UWS (group 1, *n* = 6) or the Lifor solution (group 2, *n* = 6). The grafts in the control group (group 3, *n* = 6) were immediately transplanted without preservation.

### 2.2. Preservation Solutions

Lifor solution (Lifeblood Medical, Inc., Adelphia, NJ) contained sugars, amino acids, salts, buffers, colloids, and lipid nanoparticles (295 ± 4 mosmol/L, pH 7.07 ± 0.01, PCO_2_  5.0 ± 0.2 mmHg, PO_2_  169 ± 2 mmHg, Na^+^  98 ± 1 mmol, K^+^  15.8 ± 0.4 mmol, and Ca^2+^  0.17 ± 0.02 mmol). UW (Bristol-Myers Squibb Company, New York, USA) solution contained phosphate, adenosine, lactobionate, raffinose, allopurinol, glutathione, buffers, and pentafraction (335 ± 4 mosmol/L, pH 7.33 ± 0.01, PCO_2_  6.7 ± 2.3 mmHg, PO_2_  167 ± 8 mmHg, Na^+^  39 ± 2 mmol, K^+^  94 ± 2 mmol, and Ca^2+^  0.08 ± 0.01 mmol).

### 2.3. Small Bowel Transplantation

After 24-hour fast with water ad libitum, swine were premedicated with ketamine (20 mg/kg) and atropine (0.06 mg/kg) and anesthesia was induced and maintained with intravenous injection of 150 mg/kg/min propofol (Disoprivan 2%, emulsion; AstraZeneca, Wedel, Germany). In donor animals, a 200 cm segment of the ileum was isolated on a pedicle of the superior mesenteric artery (SMA) and the superior mesenteric vein (SMV) following a midline laparotomy. After cold flushing (0~4°C) via the artery with heparinized preserving liquid, the harvested intestinal segment was preserved in an icebox for 9 hours. At the end of the preservation, the graft was allotransplanted with anastmoses of SMA and SMV to the right external iliac artery and vein of recipient, respectively, in an end-to-end fashion. Both ends of the graft intestine were exteriorized through the bilateral abdominal walls as a Thiry-Vella loop.

All animals were allowed to drink only water ad libitum during the period from postoperative day 1 to day 3. FK506 (Astellas Ireland Co., Ltd., Ireland) was administered intravenously (0.1 mg/kg per day) for immunosuppression. In addition, they were intravenously administered maintenance infusion therapy of heparin (100 U/kg per day) and antibiotics. Full oral feeding, including solid food, was started on postoperative day 4. At the end of experiment, all animals were euthanized by anesthesia overdose, followed by an intravenous injection of saturated potassium chloride.

### 2.4. Histological Examination

At the end of the preservation (PRE), 1 h after ischemia-reperfusion (IRI), and day 7 after transplantation (POD7), graft biopsy specimens of terminal harvested ileum were taken and placed in formalin for standard processing (hematoxylin and eosin staining). Histopathology tissue sections, stained with hematoxylin and eosin (H&E), were evaluated by light microscopy. Histological injury of the intestinal samples was quantitatively evaluated according to the scoring system of Regner et al. in a blinded manner [12]. The grades are as follows: 0, normal mucosa; 1, subepithelial Gruenhagen's space (oedema) at the apex of villi; 2, extension of subepithelial space with moderate epithelial lifting; 3, large subepithelial space and extensive epithelial lifting with occasional denuded villi tips; 4, denuded villi with dilated capillaries; and 5, lamina propria disintegration, hemorrhage, and ulceration.

### 2.5. Adenosine Triphosphate (ATP) Concentration

After 9-hour preservation, graft samples of terminal harvested ileum were snap frozen in liquid nitrogen and preserved below −80°C until later analysis. Frozen samples were weighed and then extracted 1 : 5 w/v in perchloric acid containing 1 mM EDTA. The precipitated protein was removed through centrifugation. Acid extracts were neutralized by the addition of 3 M KOH/0.4 M Tris/0.3 M KCl and then recentrifuged. Aliquots of neutralized extracts were immediately processed via standard enzyme-linked metabolite assays. And then spectrophotometric analysis was performed to measure the absorbance of NADH at 340 nm, providing quantification of ATP. Values are reported as micromoles per gram protein. Protein was measured according to Chiu et al. [13].

### 2.6. Intestinal Absorption Tests

Maltose absorption test was performed on postoperative day (POD) 7 to evaluate intestinal absorptive function [14, 15]. Maltose (1 g/kg) dissolved in 10 mL of normal saline was injected into the graft intestinal lumen, and the ileumstomy was occluded by a Foley catheter. Blood glucose levels were measured in femoral blood collected every 15 minutes for 120 minutes using a glucose meter.

### 2.7. Statistical Analysis

Nonparametric test was used for statistical analysis by using SPSS 16.0 (SPSS, Inc.) Software. All data are expressed as mean ± SEM and a *p* value < 0.05 was considered statistically significant.

## 3. Results

### 3.1. Survival

All animals of the three groups survived without serious complications until day 7 of the graft assessment.

### 3.2. Histology

**Figure 1:**
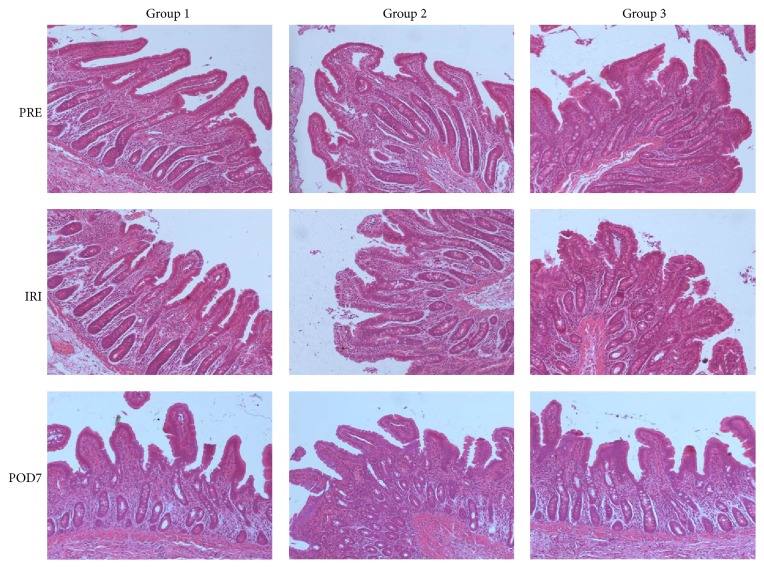
Morphologic changes of small bowel mucosa (HE ×100).

As shown in Figure 1, slight injured mucosal epithelium, edema, and an infiltration of necrotic epithelial cells could be found in the intestinal villi and the gap between epithelial cells slightly increased after 9-hour cold preservation, and histological specimens taken 1 h after reperfusion showed loss of villous tissue with hemorrhage and substantial submucosal edema, which was similarly seen in groups 1 and 2 (*p* > 0.05). At day 7 after transplantation, no pathologic findings of even mild acute rejection were observed in any grafts that were evaluated and histological studies showed almost normal structure of the small intestinal mucosa in all groups.

**Figure 2:**
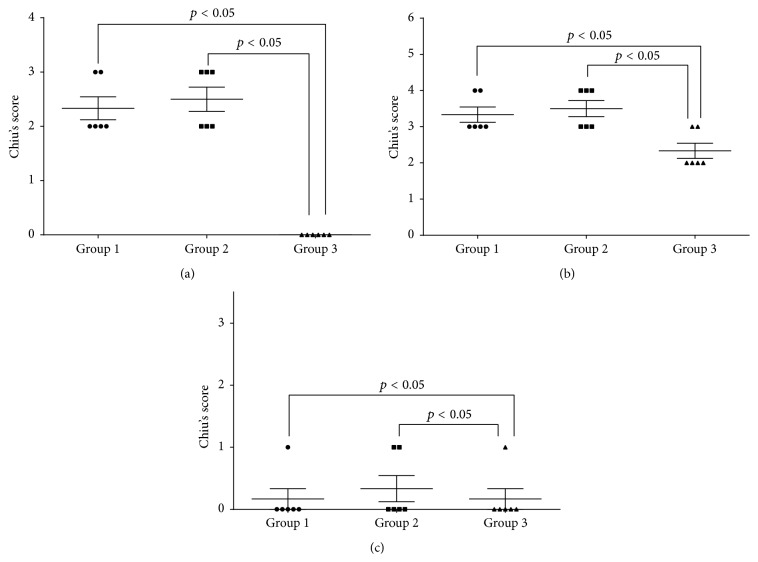
Intestinal injury scores. (a) at the end of the preservation; (b) 1 h after ischemia-reperfusion; (c) day 7 after transplantation.

Based on the Chiu's scoring system, significantly higher intestinal injury scores were determined in groups 1 and 2 after preservation and 1 h after reperfusion compared with group 3 (*p* < 0.05) (Figure 2).

### 3.3. Intestinal Mucosal ATP Concentration

**Figure 3:**
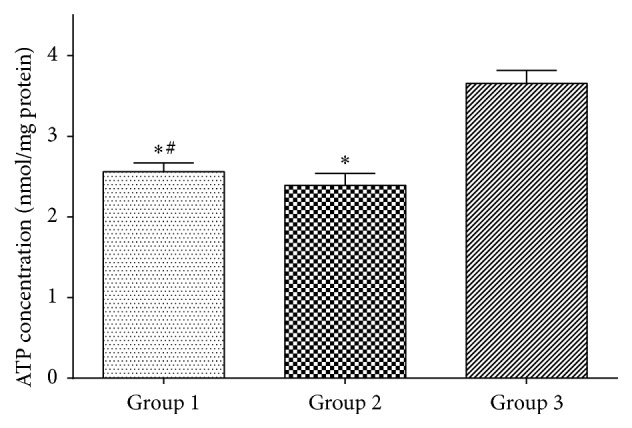
Intestinal mucosal ATP concentration at the end of preservation (^*∗*^ *p* < 0.05 versus group 3; ^#^ *p* > 0.05 versus group 2).

The tissue ATP concentration of the graft after CS is regarded as one of the most important parameters reflecting the organ viability. Although the tissue ATP concentrations of groups 1 and 2 were significantly decreased compared with group 1 (*p* < 0.05), no significant difference was noted between groups 1 and 2 (*p* > 0.05, Figure 3).

### 3.4. Intestinal Absorption Tests

**Figure 4:**
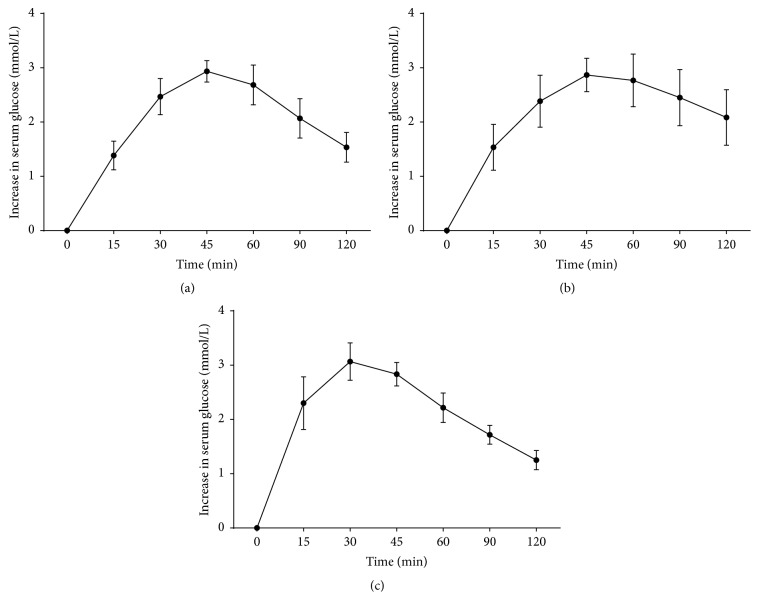
Maltose absorption test at day 7 after transplantation.

Figure 4 showed curves of increase in serum glucose level from the baseline. Serum glucose levels in group 2 were comparable to those in group 1 at all measurement points. Serum glucose curves showed a peak level at 30 min in group 3. In contrast, in groups 1 and 2 the peak of the serum glucose curve was delayed to 45 min. Although area under the curve for 30 minutes of groups 1 and 2 was significantly decreased compared with group 1 (*p* < 0.05), no significant difference was noted between groups 1 and 2 (*p* > 0.05, Table 1).

## 4. Discussion

ITx has become the therapy of choice for patients with IF as management strategies evolve in recent years, but unique clinical problems still remain, including the lack of a potent preservation strategy for the small bowel [16]. Graft viability prior to implantation is a key factor in the outcome after organ transplantation [17, 18]. The use of UWS for the small bowel remains inferior as compared with other solid organs such as the kidney, liver, and pancreas, which limits the clinical success of intestinal transplantation [5–7]. Therefore, actively seeking an optimal preservation solution and technique appropriate for IP is still the hotspot in current area of transplantation.

Lifor, a nanoparticle solution containing amino acids, salts, sugars, and other additives, as a novel organ preservation solution was firstly assessed by Stowe et al. in a model of low-flow perfused swine hearts [8, 9]. Under ambient air and temperature conditions, perfusion with Lifor solution for 10 h and 20 h provided superior protection against cardiac injury compared with UWS perfusion. Recently, Stowe et al. estimated Lifor in a porcine model of renal perfusion at room temperature [10]. In comparison to hypothermic or room temperature machine perfusion with UWS, kidneys perfused with Lifor at room temperature demonstrated remarkable improvements in output flow and intrarenal resistance. Soon afterwards, Gage et al. provided further evidence of the efficacy of Lifor in warm ischemia and meanwhile demonstrated that Lifor solution mitigated renal IR under cold storage conditions compared with UWS [11].

Notably, Lifor has been reported to contain cellular nutrients, buffers, salts, and lipid nanoparticles, but the proprietary nature of Lifor solution precludes a detailed comparison to UW [8, 9]. Nevertheless, there are several reported differences between UW and Lifor that may influence postischemic organ function. The K^+^ content of UW is dramatically higher than that of Lifor. Although high K^+^ solutions can effectively maintain intracellular ion balance during organ preservation, the high K^+^ concentrations can subsequently impair reperfusion by promoting vasoconstriction [19]. Additionally, compared with Lifor solution, UWS is highly viscous and has been associated with red blood cell aggregation and microcirculatory abnormalities in the models of organ transplantation [20, 21]. Taken together, these differences between Lifor and UW may explain the superior protection of Lifor in these studies.

In contrast with solid organ, the intestinal mucosa is much more vulnerable to injury resulting from ischemia [12, 22]. In addition, the effect of Lifor on intestinal injury was not explored previously. Now we performed the study to determine whether Lifor solution was more effective than UW in preserving small bowel under cold storage conditions in the model of swine segmental small bowel autotransplantation. The results of the histologic examination indicated that the damage degree of intestinal mucosa has no significant difference at the end of preservation between UW and Lifor group. Although the damage degree of tissue is associated with graft viability before transplantation, the graft viability cannot be well predicted after transplantation [17]. Instead, tissue ATP concentration is considered a surrogate of graft viability, which can well predict graft function after transplantation [23–25]. The reduction of ATP concentration during ischemia is proportional to the degree of tissue injury and intestinal mucosal hyperpermeability after IR [23–25]. Therefore, ATP concentration is thought to be a determinant of intestinal barrier function. And the results revealed that no significant differences in the concentration of ATP between the two groups were observed.

In this study, we prolonged the preservation period up to 9 h and tested graft viability by assessing the ATP concentration, histologic findings, and intestinal absorptive function after transplantation. At 9 h, the cold Lifor storage succeeded in preserving the graft according to the changes in the parameters obtained in comparison with UWS. With the extension of preservation time, the reduction of ATP concentration is coordinated with the damage degree of intestinal tissue injury, and no significant difference was found between UW and Lifor groups.

In summary, the effect of short-term cold storage of canine small bowel with Lifor solution is similar to that of UW solution. In addition, this study provides additional rationale for further exploration of Lifor as an alternative preservation solution in small bowel transplantation.

## Figures and Tables

**Table 1:** Maltose absorption test.

Group	Time to the peak level (min)	Area under the curve
1	45 ± 3.87^*∗*#^	2.62 ± 0.15^*∗*#^
2	47.5 ± 4.61^*∗*^	2.73 ± 0.26^*∗*^
3	32.5 ± 2.5	3.83 ± 0.25

Note: ^*∗*^ *p* < 0.05 versus group 3; ^#^ *p* > 0.05 versus group 2.
